# Resistance Training Safety during and after the SARS-Cov-2 Outbreak: Practical Recommendations

**DOI:** 10.1155/2020/3292916

**Published:** 2020-09-23

**Authors:** Paulo Gentil, Claudio Andre Barbosa de Lira, Daniel Souza, Alfonso Jimenez, Xian Mayo, Anna Luiza de Fátima Pinho Lins Gryschek, Erica Gomes Pereira, Pedro Alcaraz, Antonino Bianco, Antonio Paoli, Julio Papeschi, Luiz Carlos Carnevali Junior

**Affiliations:** ^1^Faculdade de Educação Física e Dança, Universidade Federal de Goiás, Goiânia, Brazil; ^2^Liga de Hipertensão Arterial, Universidade Federal de Goiás, Goiânia, Brazil; ^3^GO fit LAB, Ingesport, Madrid, Spain; ^4^Advanced Wellbeing Research Centre, Sheffield Hallam University, Sheffield, UK; ^5^Centre for Sport Studies, King Juan Carlos University, Madrid, Spain; ^6^Departamento de Enfermagem em Saúde Coletiva da Escola de Enfermagem da Universidade de São Paulo, São Paulo, Brazil; ^7^Research Center for High Performance Sport, UCAM, Murcia, Spain; ^8^Faculty of Sport Sciences, UCAM, Murcia, Spain; ^9^Department of Psychology, Educational Science and Human Movement, University of Palermo, Palermo, Italy; ^10^Department of Biomedical Sciences, Physiological Laboratory, University of Padova, Padova, Italy; ^11^Instituto Valorize, Vitória, Brazil; ^12^Centro Universitário UniFMU, São Paulo, Brazil

## Abstract

In December of 2019, there was an outbreak of a severe acute respiratory syndrome caused by the coronavirus 2 (SARS-CoV-2 or COVID-19) in China. The virus rapidly spread into the whole world causing an unprecedented pandemic and forcing governments to impose a global quarantine, entering an extreme unknown situation. The organizational consequences of quarantine/isolation are absence of organized training and competition, lack of communication among athletes and coaches, inability to move freely, lack of adequate sunlight exposure, and inappropriate training conditions. The reduction of mobility imposed to contain the advance of the SARS-Cov-2 pandemic can negatively affect the physical condition and health of individuals leading to muscle atrophy, progressive loss of muscle strength, and reductions in neuromuscular and mechanical capacities. Resistance training (RT) might be an effective tool to counteract these adverse consequences. RT is considered an essential part of an exercise program due to its numerous health and athletic benefits. However, in the face of the SARS-Cov-2 outbreak, many people might be concerned with safety issues regarding its practice, especially in indoor exercise facilities, such as gyms and fitness centers. These concerns might be associated with RT impact in the immune system, respiratory changes, and contamination due to equipment sharing and agglomeration. In this current opinion article, we provide insights to address these issues to facilitate the return of RT practices under the new logistical and health challenges. We understand that RT can be adapted to allow its performance with measures adopted to control coronavirus outbreak such that the benefits would largely overcome the potential risks. The article provides some practical information to help on its implementation.

## 1. The Problem

Resistance training (RT) is a popular exercise mode and has been recommended as an essential part of a physical exercise program by several important associations [1, 2]. Its benefits, largely mediated by strength gains, culminate in reductions in mortality rates in different populations [3–7] and expand to several areas such as blood pressure control [8], improved bone mineral density [9], depression management [10], cancer treatment [11], blood glucose management [12], and weight management [13], among others.

In December of 2019, there was an outbreak of a severe acute respiratory syndrome caused by the coronavirus (SARS-CoV-2 or COVID-19) in Wuhan, Hubei Province, China. The virus rapidly spread across the country and then into the whole world [14], causing an unprecedented pandemic, which forced governments to impose an almost global quarantine. At the beginning of 2020 (January–May), the whole world, including the world of sports, entered in an extreme and unknown situation, where gradually, all sports competitions were postponed and any organized training or practice was banned [15, 16]. In the face of the SARS-Cov-2 outbreak, most countries recommended social distancing and isolation and imposed severe restrictions to the exercise facilities where RT is commonly performed, such as gyms and fitness centers. In many countries, exercise facilities were prohibited from opening. Moreover, the reduction of mobility imposed by the challenges of containing the advance of the pandemic (social isolation and circulation restrictions) may have a negative impact on the physical fitness leading to muscle atrophy, progressive loss of muscle strength, and reductions in neuromuscular and mechanical abilities [17, 18].

After the initial moment, many national health authorities have allowed the functioning of exercise facilities; notwithstanding, this is occurring under strict regulations. These measures commonly involve social distancing, control of the number of people inside exercise facilities, use of protective masks, sterilization of materials, and avoidance of contaminations through touching and handling objects [19, 20].

Although these measures are important to control virus dissemination, it might be a barrier for RT practice, since many people could find it challenging to train under these regulations. Another possible barrier is that, even with safety measures, many people might feel unsafe to perform RT in exercise facilities. Therefore, it is important to scientifically address these possible barriers and propose practical solutions to help health professional to stimulate the reestablishment of healthy habits by the general public.

This issue is important, because around 30-40% of world population did not meet the minimum amount of exercise preconized for the maintenance and improvement of health [21] and because noncommunicable diseases related to physical inactivity are responsible by 5.3 million deaths each year [22]. Based on this, the present article is aimed at proposing an evidence-based and practical discussion about the topic and providing technical elements that might help to put RT into practice after allowance by the national health agencies.

## 2. Measures to Control Contamination Risk

COVID-19 is a respiratory infection caused by the SARS-CoV-2 virus, which is a new form of coronavirus [23]. The virus is transmitted directly travelling through the air to the mouth, nose, or eyes (T zone) or by objects or surfaces touched by a recipient who then touches the T zone [24]. A previous study evaluated the stability of SARS-CoV-2 and SARS-CoV-1 in aerosols and on various surfaces and reported that SARS-Cov-2 remained in the air for 3 hours [25]. Regarding solid surfaces, evidences suggest that SARS-Cov-2 can remain on metal, cardboard, glass, or plastic for more than a week [25, 26]. For this reason, special care must be taken by personnel that work or train in exercise facilities. Use of masks and constant sterilization of materials (machines, bars, and dumbbells) should be mandatory.

While the exact disinfection practices outside health care settings are currently unknown, it is recommended that exercise facilities adopt cleaning and disinfection procedures previously recommended to prevent and control the spread of pathogens [24, 26–28]. Products such as 62-71% ethanol, 0.5% hydrogen peroxide, or 1% sodium hypochlorite can be effective in eliminating the virus on solid surfaces [27]. Therefore, they should be readily available for everyone entering or remaining in exercise facilities. Spraying individuals with disinfectants (such as in a tunnel, closet, or chamber) is not recommended under any circumstances [27].

It is also necessary to reinforce hygiene measures, like using discarded tissues when coughing or sneezing, avoiding autoinoculation by not touching T zone, washing/disinfecting hands thoroughly, and maintaining physical distance [24]. It should be recommended to maintain a distance of at least 1 meter between people (one person per 4 to 6/m^2^), avoid direct physical contact with other people (for example, hugging, touching, and shaking hands), and promote strict control over external access (temperature measurement with a remote thermometer, observance of the use of masks, and hand disinfection) [29]. Some supportive measures should be environment restructuring (separate equipment, create ground marks, deactivate digital access), provide hand sanitizers or washbasins thoroughly inside facilities, schedule specific cleaning times, schedule training times, encourage the use of individual bottle, and observe air conditioning specification with respect to air exchange.

## 3. Immune System and Exercise

The immune system is important to protect against the negative consequences of opportunistic infections through the coordinated functions of many cells [30]. Therefore, it is important to preserve or improve its function during a virus outbreak. While the specific effects of RT on immune function are not well understood [31], evidences show that there might be either immune surveillance or immunodepression in response to exercise, depending on how it is performed [32–34]. Interestingly, immune depression and illness seem to be more common in people involved with endurance activities [35] when compared to strength and power activities [36–39], which might be a positive point to RT [40].

The association between exercise, the immune system, and risk of illness seems to follow a J-shaped curve [32–34]. Body defenses improve with moderate amounts of physical exercise and decrease with excessive or low amounts of exercise [32–34]. Consequently, the risk of infection is higher in sedentary individuals and also in those engaged in high volume/intensity of physical exercise, such as endurance athletes. This complex relation seems to be related to many factors, becoming more negative with increased training volume [41–43] and higher energy expenditure [44, 45], and it is also modulated by the physiological responses to exercise [30]. For this reason, it might be advisable to adopt a reduced total training volume/duration (<1 hour) in order to preserve immune function and prevent illness [33, 42]. With that in mind, the adoption of time-efficient RT approaches might be attractive, especially if we consider those training sessions lasting few minutes can be efficient in promoting muscle strength and size gains in different populations [46, 47]. For example, untrained young and older adults can benefit from minimal dose RT protocols that involve two sets of 3 to 4 exercises performed once or twice per week [48–51].

Another important aspect to consider is the physiological response to physical exercise, since elevations in cortisol, epinephrine, and sympathetic modulation seem to be related to exercise-induced immunosuppression [30, 32]. In agreement with this, previous studies have shown that elevated metabolic stress and cortisol levels might be associated with immunosuppression in response to RT [52–54]. Therefore, it might be interesting to avoid such response to preserve immune function during a virus pandemic. In this regard, previous studies have shown that RT protocols with few numbers of repetition (≤6 repetitions) and large interval between sets (≥3 minutes) result in less pronounced increases in lactate and cortisol levels [55–57], making them possibly more recommended when trying to avoid the harms of a viral infection. Furthermore, considering that glucose is the main fuel of immune cells [58], the performance of low-volume RT with few repetitions would be less glycolytic dependent [59] and it could prevent the concurrency for energy substrate and subsequently transitory impairment on immune competency.

## 4. Respiratory System and Exercise

One of the main sources of SARS-Cov-2 contamination is through the respiratory system of the infected people [41]. Based on this, one of the recommended measures to avoid contaminations is the use of protective masks [23, 29]. With regard to exercise, controlling respiratory responses to exercise is particularly important to avoid virus dissemination and also to alleviate the discomfort of training while using protective masks.

When compared to training without protective barriers, the use of any type of masks reduces the flow of air to the lungs, causing repercussions such as less oxygen supply to the lungs and consequently less supply to the muscles, which makes training difficult. In this case, an increase in respiratory rate is expected and some symptoms may appear, such as dizziness, shortness of breath, and decrease in performance [60–62]. Notwithstanding, it has been demonstrated that a period of adaptation and familiarization with the use of protective masks can reduce or even eliminate such symptoms and discomfort [63].

One possible advantage of RT is that it usually promotes less pronounced cardiorespiratory responses (i.e., increase in pulmonary ventilation and oxygen consumption) when compared to aerobic exercise [64–66]. To further control respiratory response, it might be important to observe the manipulation of RT variables. Previous studies reported higher values of oxygen consumption and pulmonary ventilation with increased volume/duration [67–69], lower rest intervals [70, 71], higher movement velocities [72–74], and higher number of repetitions [75, 76], especially when effort is matched. Therefore, in order to reduce the cardiorespiratory stress and consequently the risk of contamination due to changes in pulmonary ventilation and the dyspnea associated with training with masks, it would be recommended to train with a lower number of repetitions, higher interval between sets, and controlled movement velocity [77]. The use of very low movement velocity that could be useful to provide a higher time under tension and lead to increasing on muscle strength and size, even when using low absolute loads, has have been shown in older [78] and young people [79, 80].

It is difficult to address the effects of safety masks in RT performance due to the lack of specific evidence on that topic. Studies involving masks that restrict breathing are controversial, with some showing no detrimental effects on performance when compared to a control condition [81], while others reported decreases in total training volume [82]. However, it is important to note that the masks used in these studies were intentionally designed to restrict air flow and oxygen availability, which might not be the case with conventional protective masks that have been used by population.

## 5. Equipment and Space

The SARS-Cov-2 might be transmitted by travelling through the air from an infected person's airways (asymptomatic or symptomatic) and also by solid surfaces, where it might stay active for several days [25, 26]. Therefore, in addition to the disinfection measures previously cited, it might be important to minimize the manipulation of objects (bars and dumbbells) to decrease the risk of contamination, which can be achieved by adequate exercise selection. RT programs usually involve many exercises, including isolated exercises for specific muscles; this would require the practitioner to manipulate many different implements, which could lead to a higher risk of contamination. However, the use of multijoint exercises seems to be sufficient to promote gains in muscle strength and size in most muscles involved in movement (i.e., biceps, gluteus, quadriceps, triceps, and biceps brachii) [83–87] and the use of isolated exercises does not seem to bring additional benefits [86, 88, 89]. Therefore, the use of a few complex exercises combined with low-volume approaches might be particularly convenient, since this will involve less manipulation of objects and reduce the need of remaining and displacing inside exercise facilities, which reduced the exposure to infection and decrease the need to constant disinfection. Moreover, this will also allow having fewer people in the exercise facility at the same time, providing better use of space and facilitating social distancing.

In same situations, it might not be possible to train multijoint exercises (e.g., barbell squat) due to difficulties in performance such as poor technique, back pain, and limited mobility. In this case, the use of single-joint exercises in an consecutive agonist/antagonist approach might be helpful to maintain the time-efficient strategy [90].

## 6. Final Considerations

The impacts of the SARS-Cov-2 pandemic on health and economic areas are major concerns for most countries. With people at home, sedentary behavior and physical inactivity may greatly increase, posing important health [91] and economic burdens [92]. Considering the relevant benefits of RT addressing the negative effects of the locking down (i.e., sarcopenia-dynapenia, increased body fat, metabolic health, and mental health), it is important to stimulate its practice as soon as possible, following the health agencies' recommendations. Although RT might be performed in different spaces and using different equipment [93], exercise facilities are still the most popular place to its practice, due to logistical and motivational reasons. Furthermore, in these places, there are specialized staff with adequate academic background to guide and advise RT practitioner to obtain best results in actual pandemic scenario. It is important to highlight that this article is not intended to confront or minimize safety and protective measures imposed by health authorities or deny the possibility of performing RT in nonspecialized facilities or without specific equipment. It only brings scientific information to support actions for those who chose or need to exercise under specific conditions.

According to the existing evidences, the use of low-volume/duration approaches and the manipulation of training variables (low number of sets, exercise choice, high rest intervals, and controlled movement velocity) might be particularly convenient, since it would help the RT programs to be safer and logistically feasible during a pandemic period and especially immediately after.

## Data Availability

All data used in the study is available under request.
